# Triglyceride is an independent predictor of type 2 diabetes among middle-aged and older adults: a prospective study with 8-year follow-ups in two cohorts

**DOI:** 10.1186/s12967-019-02156-3

**Published:** 2019-12-03

**Authors:** Jing Zhao, Yuan Zhang, Fengjiang Wei, Jiani Song, Zhi Cao, Chen Chen, Kai Zhang, Shuzhi Feng, Yaogang Wang, Wei-Dong Li

**Affiliations:** 1 Department of Genetics, College of Basic Medical Sciences, Tianjin Medical University, 22 Qixiangtai Road, Heping District, Tianjin, 300070 People’s Republic of China; 2 College of Public Health, Tianjin Medical University, 22 Qixiangtai Road, Heping District, Tianjin, 300070 People’s Republic of China; 3 Tianjin General Hospital, Tianjin Medical University, 154 Anshan Road, Tianjin, 300052 People’s Republic of China

**Keywords:** Triglyceride, Type 2 diabetes mellitus, Dynamic cohort study, Survival analysis

## Abstract

**Background:**

Although there is abundant evidence indicating the connection between triglyceride and type 2 diabetes mellitus (T2DM), few reports or cohort studies confirm that high TG concentration may predict the incidence of T2DM independently. Thus, we studied the association between triglyceride (TG) and T2DM in a male-dominated, middle and older aged cohort, Tianjin General Hospital Cohort. And we further verified our results in the China Health and Retirement Longitudinal Study (CHARLS).

**Methods:**

We conducted an 8-year retrospective cohort study (2009–2017) with 7241 participants who were free from T2DM at baseline. Three groups were constructed based on baseline TG levels (normal, borderline-high, and high). We used a Cox proportional hazards model to evaluate the relationship between TG and T2DM after adjusting for possible risk factors. A Kaplan–Meier survival analysis was performed to compare the incidence of T2DM among subjects in each TG group. We also tested the association between TG and T2DM in the CHARLS cohort.

**Results:**

In Tianjin General Hospital Cohort, 7241 participants (male 75.8%, female 24.2%) were included, mean age was 61.49 ± 13.85 years at baseline. The cumulative incidence of T2DM in our cohort study was 8.6% (9.2% in men and 6.6% in women). Compared with the normal TG group, the hazard ratios in the borderline and high group were 1.30 (95% *CI* 1.04–1.62) and 1.54 (95% *CI* 1.24–1.90). The Kaplan–Meier survival analysis indicated that higher TG levels may predict higher onset of T2DM. These results were verified in the CHARLS cohort, the hazard ratio with T2DM (95% *CI*) for logTG was 3.94 (2.64–5.87).

**Conclusions:**

Our findings suggest that the TG level may be an independent risk factor and predictor for T2DM.

## Background

Diabetes mellitus (DM) affects 11.6% of the Chinese adult population [1]. It is predicted that there will be a 69% increase in prevalence of adult diabetes mellitus in developing countries between 2010 and 2030 [2]. DM patients have higher risks of cardiovascular disease, chronic kidney disease, cancer, and even death [3–5]. Thus, it is important to identify individuals who have higher risks for diabetes to facilitate early prevention and treatment.

Triglycerides (TGs) are the most abundant lipid in human adipose tissue. Classification of TG level by NCEP Guide in 2001 is: desirable < 1.7 mmol/L (150 mg/dL), borderline-high 1.7–2.25 mmol/L (150–199 mg/dL), high 2.26–5.6 mmol/L (200–499 mg/dL) and very high ≥ 5.6 mmol/L (500 mg/dL). In the United States, 31% adults have a TG level ≥ 1.7 mmol/L, 16.2% of adults exhibit a high (≥ 2.26 mmol/L) TG level, while 1.1% have very high (≥ 5.6 mmol/L) TG [6]. Shown by a number of epidemiological studies, TG level was linearly correlated with the risk of T2DM [7–9]. Furthermore, a study indicated that among many lipid indexes which might be associated with T2DM, TG was the most significant [10]. Until now, some studies have been aimed at the relationship between TG level and T2DM in young men or women [11, 12]. There are also some cross-sectional studies showing the link between TG and T2DM [13, 14]. But there are few reports or cohort studies which confirm that high TG concentration may predict the incidence of T2DM independently after adequately adjusting for potential confounders.

We collected a dynamic cohort, Tianjin General Hospital Cohort, consisting mainly of senior citizens who had regular physical examinations each year from 2009 to 2017 in Tianjin, China. By using Cox regression analysis, Kaplan–Meier survival analysis and other statistic method, We aim to reveal the association between TG and T2DM. We furthermore validated our result in another cohort, the China Health and Retirement Longitudinal Study (CHARLS).

## Materials and methods

### Study participants

We collected 8956 subjects from Tianjin General Hospital Cohort who had annual comprehensive physical examinations from 2009 to 2017. Among these individuals, 1715 subjects were excluded due to prevalent T2DM or missing data in TG and fasting plasma glucose levels. A total of 7241 subjects were included in this study. All participants gave written informed consent prior the study, and the protocol was authorized by the Human Ethics Committee of Tianjin Medical University.

The China Health and Retirement Longitudinal Study (CHARLS) is a nationally representative longitudinal survey, investigating people of 45 years of age or older in China. We selected 17,500 people in CHARLS from 2011 to 2015. Among these people, 5653 of them who lacked data of follow-up or suffered from DM were excluded. 2257 participants missed FPG or other covariates were excluded. Finally, a total of 9590 subjects were included to validate our result.

### Data collection

Information of all the participants’ physical examination are collected every year, from 2009 to 2017. Including glucose and lipid metabolism related index and some function related data, such as renal and liver function. All the measurements were performed by well-trained nurses or doctors. Body mass index (BMI) was calculated as weight in kilograms divided by height in meters squared. Blood pressure was measured after sitting for at least 5 min by standard mercury sphygmomanometer, and the average of two values reported. Blood samples were drawn from the antecubital vein after an overnight fast. Venous blood samples were used to measure serum uric acid (SUA) levels, lipid profiles (total cholesterol, TC; TG), fasting glucose, renal function (estimated glomerular filtration rate), liver function (alanine aminotransferase), hyperuricemia. TC and TG concentrations were measured by enzymatic calorimetric tests.

Information about CHARLS were downloaded from http://charls.pku.edu.cn/zh-CN after having permission.

### Definition

T2DM was defined in subjects as fasting plasma glucose ≥ 7.0 mmol/L or subjects described T2DM history, and/or if the subject is currently using antidiabetic medication. First occasion of documented T2DM per subject was recorded as the index event to calculate incidence rates. Hypertension was defined as the subject having a systolic blood pressure of greater than 140 mm/Hg and/or a diastolic blood pressure of greater than 90 mm/Hg [15]. When an estimated glomerular filtration rate (eGFR) was observed to be < 60 mL/min/1.73 m^2^, the subject was defined to have chronic kidney disease. Hyperuricemia was defined when serum uric acid (SUA) ≥ 7.0 mg/dL (420 μmL/L) in males and SUA ≥ 6.0 mg/dL (360 μmL/dL) in females.

### Exposure data

According to the TG concentration of participants in the baseline. Three groups were categorized in both Tianjin General Hospital Cohort and CHARLS by TG level: normal < 1.7 mmol/L, borderline high 1.7–2.25 mmol/L, and high ≥ 2.26 mmol/L.

### Statistical analyses

The data for continuous variables used mean ± SD to describe while categorical variables used percentages (%). First, we analyzed the basic characteristics in different groups at baseline in Tianjin General Hospital Cohort. In order to explore the relationship between TG and T2DM, a Cox proportional hazards model was established to evaluate hazard ratios (HR) and 95% confidence intervals (*CI*) of the incidence of T2DM by TG levels. As TG level in population is not meet Gaussian distribution, we take logTG an index to Cox proportional hazards model to study the association between TG and T2DM. Based on TG concentration grouping, we used a Kaplan–Meier survival analysis to compare cumulative incidences of T2DM among the three groups, to see if high TG level accompany high cumulative incidence of T2DM. Further stratified analyses get HR and *CI* for the incidence of T2DM which were conducted to defined in various subgroups: age group (< 60, ≥ 60 years), sex (male, female), hypertension (yes, no), BMI (< 24, 24–27.9, ≥ 28 kg/m^2^), eGFR (< 60, ≥ 60 mL/min/1.73 m^2^), hyperuricemia (yes, no). Results of stratified analyses are tested by log-rank. All statistical analyses were performed using SPSS statistical software for Windows, version 17.0 (SPSS Inc., Chicago, IL, USA).

## Results

The mean age of the 7241 participants was 61.49 ± 13.85 years, with 5492 men (75.8%; 67.75 ± 12.98 years) and 1749 women (24.2%; 67.1 ± 12.18 years) at baseline. Table 1 showed the baseline characteristic of subjects in Tianjin General Hospital Cohort. Our results revealed that the individuals who had higher TG levels were likely to have higher FPG, BMI, SUA, ALT, and blood pressure.

**Table 1:** Baseline characteristics of subjects in Tianjin General Hospital Cohort

Characteristics	Normal (< 1.7)	Borderline (1.7–2.25)	High (≥ 2.26)	*P*-value*
Male (%)	3639 (73.9%)	898 (77.3%)	955 (82.5%)	< 0.001
Age (years)	62.05 ± 14.27	61.76 ± 13.12	58.83 ± 12.36	< 0.001
BMI (kg/m^2^)	24.16 ± 3.14	25.36 ± 2.29	26.01 ± 2.78	< 0.001
Hypertension	2015/4569 (40.9)	550/1065 (47.3)	539/1041 (46.5)	< 0.001
FPG (mmol/L)	5.08 ± 0.64	5.16 ± 0.66	5.17 ± 0.66	< 0.001
eGFR-mL/min/1.73^2^	88.72 ± 19.60	86.57 ± 17.45	87.26 ± 23.18	< 0.001
TG (mmol/L)^†^	1.09 ± 0.32	1.94 ± 0.16	3.39 ± 1.67	< 0.001
TC (mmol/L)	4.76 ± 0.83	5.05 ± 0.86	5.21 ± 0.89	< 0.001
Urine dipstick result ≥ 1 + protein-no./total no (%)	140/4290 (3.2)	50/1058 (3.8)	41/1014 (4.0)	0.275
Hyperuricemia	541/4919 (11)	234/1158 (20.1)	347/1158 (30)	< 0.001
SUA (μmol/L)	319.74 ± 76.37	351.90 ± 75.85	375.08 ± 79.58	< 0.001
ALT (IU/L)	21.75 ± 17.69	25.92 ± 15.25	28.59 ± 12.15	< 0.001

Date are expressed as mean ± SD, *n* (%)

*BMI* body mass index, Hypertension, *FPG* fasting plasma glucose, *TG* plasma triglyceride level, *TC* total cholesterol, *eGFR* estimated glomerular filtration rate, hyperuricemia, *SUA* serum uric acid, *ALT* alanine aminotransferase

*P < 0.05 was considered statistically significant

^†^Grouped based on TG level: normal < 150 mg/dL; borderline high 150–199 mg/dL; high 200–499 mg/dL

The cumulative incidence of T2DM in Tianjin General Hospital Cohort was 8.6% (9.2% in males; 6.6% in females; χ^2^ = 11.254, *P* = 0.001) during the 8-year follow-up. Table 2 showed that logTG, hypertension, BMI, HUA, and sex were significantly associated with T2DM, while logTG was the most significant. The hazard ratio (95% *CI*) for logTG was 2.58 (1.87–3.57), while for hypertension, BMI, hyperuricemia, and female sex, these ratios were 1.88 (1.59–2.22), 1.11 (1.09–1.14), 1.34 (1.10–1.63), and 0.689 (0.56–0.84). We tested the association between TG and T2DM with the China Health and Retirement Longitudinal Study (CHARLS). Table 2 showed that the hazard ratio with T2DM (95% *CI*) for logTG was 3.94 (2.64–5.87). Hypertension, BMI, HUA, and sex were also associated with the risks of T2DM; the hazard ratios (95% *CI*) were 1.37 (1.11–1.69), 1.10 (1.07–1.13), 0.64 (0.42–0.96), and 0.81 (0.65–1.00), respectively.

**Table 2:** Hazard ratios of TG levels for T2DM in the Tianjin General Hospital Cohort and in the CHARLS

	Tianjin General Hospital Cohort HR (95% *CI*)^†^	CHARLS HR (95% *CI*)^†^	*P*-value*	
			Tianjin General Hospital Cohort	CHARLS
logTG	2.58 (1.87–3.57)	3.94 (2.64–5.87)	< 0.001	0.001
Female	0.69 (0.56–0.84)	0.81 (0.65–1.00)	< 0.001	0.046
Age (years)	1.026 (1.02–1.03)	1.02 (1.00–1.03)	< 0.001	0.005
BMI (kg/m^2^)	1.11 (1.09–1.14)	1.10 (1.07–1.13)	< 0.001	< 0.001
Hypertension	1.88 (1.59–2.22)	1.37 (1.11–1.69)	< 0.001	0.004
Hyperuricemia	1.34 (1.10–1.63)	0.64 (0.42–0.96)	< 0.001	0.033

*TG* plasma triglyceride level, *BMI* body mass index

*P < 0.05 was considered statistically significant

^†^Each time the value of hazard ratios changes by one unit, the risk doubles

Our findings suggest that the correlation between TG and T2DM remained in all three models both in Tianjin General Hospital Cohort and CHARLS (Table 3). In Tianjin General Hospital Cohort, compared with the first group, in an unadjusted model, the hazard ratios for T2DM in the second and third groups were 1.42 (1.16–1.74) and 1.65 (1.36–2.00), respectively. After adjusting for age and sex, the hazard ratios in the second and third groups were 1.45 (1.18–1.78) and 1.75 (1.44–2.12). After further adjustment, the association remained significant. In model 3, the hazard ratios (95% *CI*) of the second and third groups versus first group were 1.30 (1.04–1.6) and 1.54 (1.24–1.90), respectively. When the TG level was analyzed as a continuous variable, the association was still pronouncedly observed in model 1–3 between high TG level and T2DM. These results in CHARLS were consistent with our results, showing that TG level is associated with T2DM. In CHARLS, it showed that the correlation between TG and T2DM remained in all three models. In an unadjusted model, compared with the first group, the hazard ratios in the second and third groups were 1.86 (1.44–2.40) and 2.76 (2.20–3.48). The hazard ratios were 1.81 (1.40–2.33) and 2.74 (2.18–3.45) after adjusting for age and sex in model 2. In model 3, the hazard ratios (95% *CI*) of the second and third groups versus first group were 1.55 (1.20–2.00) and 2.20 (1.73–2.80), respectively.

**Table 3:** Prospective analysis of associations between baseline triglyceride levels and incident T2DM in the Tianjin General Hospital Cohort and in the CHARLS

	Tianjin General Hospital Cohort HR (95% *CI*)	CHARLS HR (95% *CI*)	*P*-value*	
			Tianjin General Hospital Cohort	CHARLS
Borderline^†^				
Model 1^a^	1.42 (1.16–1.74)	1.86 (1.44–2.40)	< 0.001	< 0.001
Model 2^b^	1.45 (1.18–1.78)	1.81 (1.40–2.33)	< 0.001	< 0.001
Model 3^c^	1.30 (1.04–1.62)	1.55 (1.20–2.00)	< 0.001	0.001
High^†^				
Model 1^a^	1.65 (1.36–2.00)	2.76 (2.20–3.48)	< 0.001	< 0.001
Model 2^b^	1.75 (1.44–2.12)	2.74 (2.18–3.45)	< 0.001	< 0.001
Model 3^c^	1.54 (1.24–1.90)	2.20 (1.73–2.80)	< 0.001	0.001

*P < 0.05 was considered statistically significant

^†^Groups were classified based on TG concentration: 1, < 150 mg/dL; 2, 150–199 mg/dL; 3, 200–499 mg/dL

^a^Model 1: unadjusted baseline values of variables

^b^Model 2: model 1 adjusted for gender and age

^c^Model 3: was further adjusted for BMI, Hypertension, hyperuricemia

**Fig. 1:**
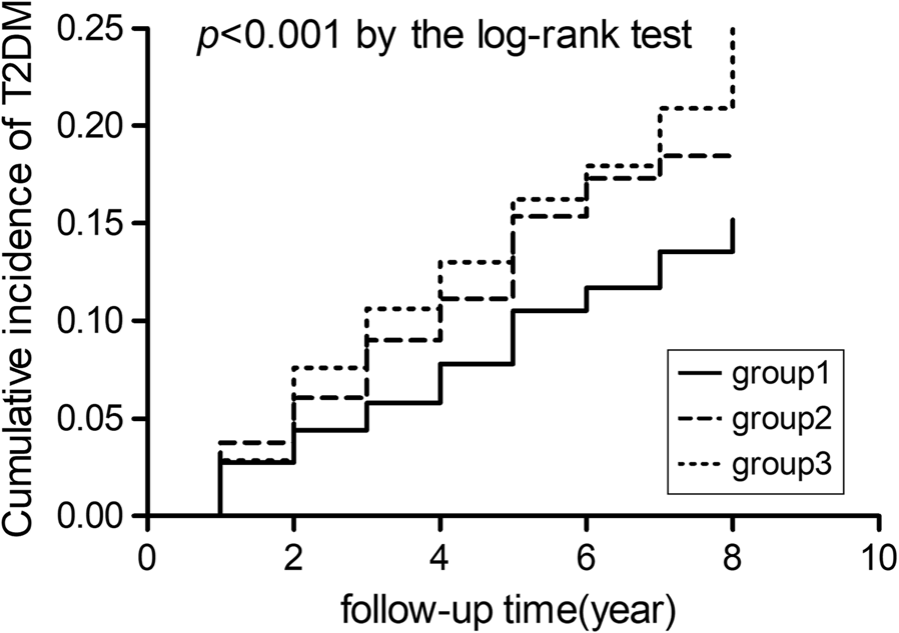
Kaplan–Meier survival analysis K–M curves for T2DM among three TG levels. ^†^Groups of TG were defined as follows: 1, < 150 mg/dL (normal); 2, 150 mg/dL-199 mg/dL (borderline high); 3, 200–499 mg/dL (high); *P-value < 0.01 for log-rank test

After testing the Kaplan–Meier survival analysis (Fig. 1) by log-rank test (*P *< 0.01), it showed that high TG levels were closely related to high incidence of T2DM in Tianjin General Hospital Cohort. The highest TG level showed the highest disease risk for T2DM among the three groups.

**Fig. 2:**
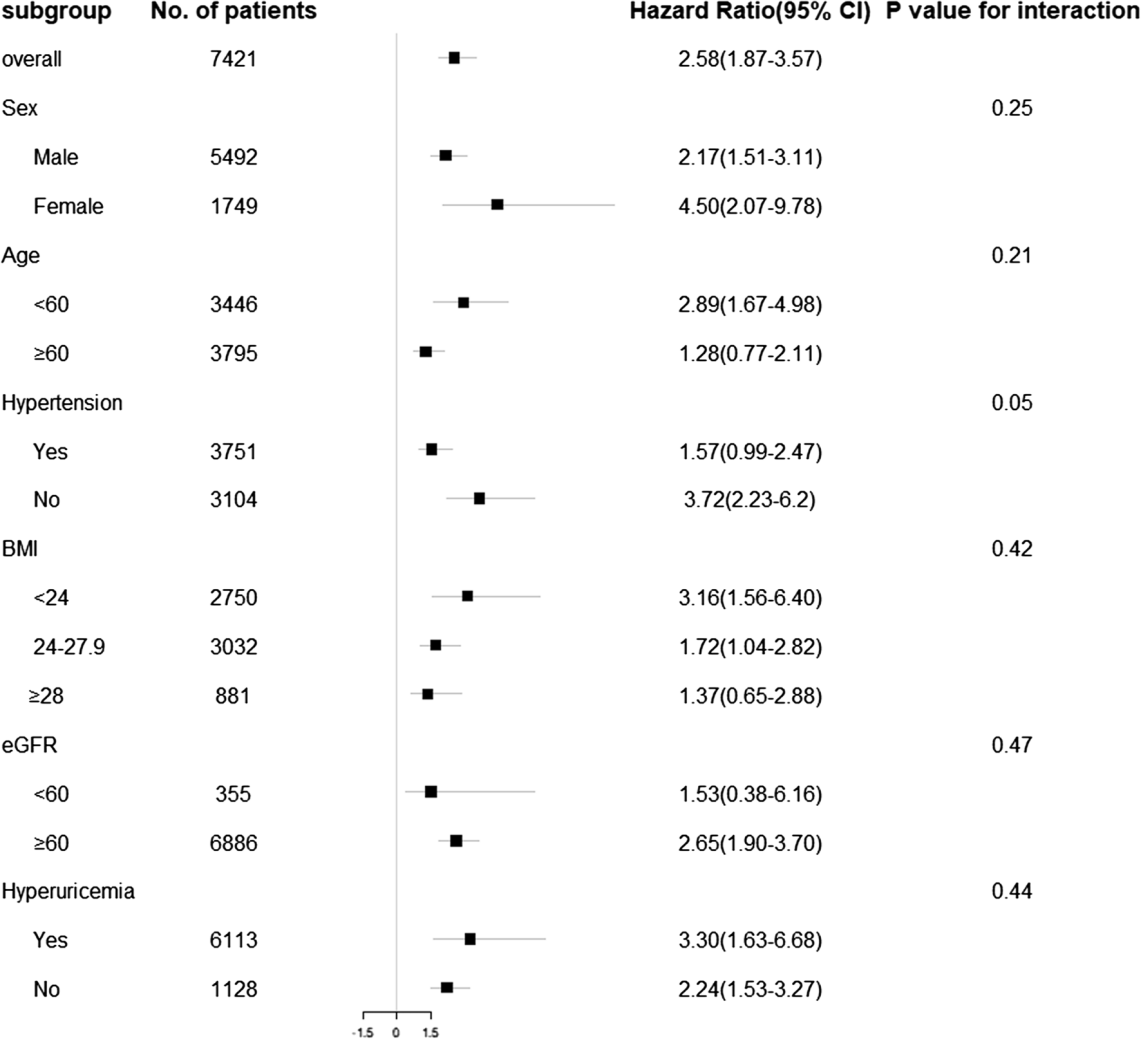
Adjusted hazard ratios (95% confidence intervals) for incidence of T2DM stratified by sex, age, hypertension, body mass index, estimated glomerular filtration rate (eGFR), and history of hypertension

Moreover, subgroup analyses (Fig. 2) indicated that the association between TG level and T2DM was more evident in people who were younger than 60 years, of normal weight (BMI < 24.0 kg/m^2^), with abnormal eGFR, with hyperuricemia, and with no hypertension.

## Discussion

Our results revealed that there was a significant linear association between TG levels and the incidence of T2DM. A higher TG level predicted higher hazard ratios of the presence of T2DM. This association was independent of age, sex, BMI, hypertension, and HUA, and it still remained significant after adjusting for other possible risk factors. The results showed that TG level may be a risk factor for T2DM.

A number of researches aimed to test whether the TG/HDL ratio could be a predictor of T2DM [10, 16, 17]. Different from previous studies, our study revealed that TG was an independent risk factor for diabetes and it might become an indicator of diabetes prediction. Our result was consistent with some earlier studies which revealed that TG level might be an influential factor for T2DM in children [18], young men [12], and women [11]. In our cohort study, we tested this relationship in an older population. Later, when this correlation (TG and T2DM) was tested in CHARLS, we were able to reach the same result. It indicated that high TG level may result in a high incidence of T2DM in the population of 45 years of age or older in China.

The cumulative incidence of T2DM in our cohort was 8.6% (9.2% in men and 6.6% in women), which was similar to the overall incidence in China. The overall pooled prevalence of diabetes was 8.3% (8.6% in men and 8.0 in women) from 2010 to 2014 [19]. But a study based on 46,239 nationally representative samples showed that the age-standardized prevalence of total diabetes was 9.7% [20]. One possible reason to explain the low prevalence of type 2 diabetes in our cohort could be attributed to the routine physical examination our subjects had each year, and may simply be relatively healthy part of the population. Another factor could be due to the likely higher social-economic status and higher income level these subjects have over the nationally representative group. A large population-based cross-sectional study in Tianjin found that people with low monthly income were associated with increased odds of T2DM [21]. It could support our results to a certain extent because the cohort we used were all from Tianjin as well.

The relationship between TG and T2DM risk was revealed in our study. Among numerous related factors, logTG has the biggest hazard ratios in diabetes mellitus. This was shown both in our original study and in CHARLS. Additionally, after controlling for confounding factors, borderline high and high TG levels were both correlated with an increased risk of T2DM. As an independent risk factor, high TG levels could be used as a guide for diabetes in clinical, which could make a more comprehensive prediction and assessment of health. People who have high TG levels are nearly 54% more likely to develop T2DM. The Kaplan–Meier survival analysis indicated that high TG levels may predict higher incidences of T2DM. In a previous cross-sectional study, the odds ratio was for T2DM was 1.87 (95% *CI* 1.29–2.70) [22]; while the odds ratio in prediabetes and T2DM was 1.96 (95% *CI* 1.42–2.69) and 3.91 (95% *CI* 2.78–5.51) in a rural Bangladeshi study [23]. Both of these studies showed TG was a risk factor for T2DM although there are significant differences in numerical value. A cohort study [24] with 8867 subjects followed up for 5.5 years had a very similar result compared to ours. After adjusting for confounding factors, the hazard ratios for T2DM in the second, third and fourth quartiles were 1.07 (0.94–1.23), 1.17 (1.03–1.34), and 1.48 (1.30–1.69) when comparing with the first quartile. It showed the correlation between TG and T2DM.

Numerous factors may lead to T2DM including both congenital elements and acquired factors, but the specific mechanism remains unclear. Adipose tissue can influence glucose and lipid metabolism as an endocrine organ while TGs are the most abundant lipid in adipose tissue [25]. The potential mechanism linking TG to T2DM might refer the free fatty (FFA) metabolic pathways. As a large amount of FFA, resistin, TNF-α, interleukin 6 and other compounds released by oversized adipose tissue can produce insulin resistance [26]. raising level of FFA produced by TGs can increase insulin resistance [27]. It has been already verified that the final outcome of insulin resistance is T2DM [28]. Furthermore, plasma FFA can enter cells easily and re-esterified for storage as TG, so raising TG levels may result in accumulation of TG [26, 29]. In both Tianjin General Hospital Cohort and CHARLS indicated higher TG level accompany high risk of T2DM. Considering lifestyle and diet can influence TG levels, the physical mechanism between TG and T2DM need a further exploration under a large sample size, long follow-up and clinical trials.

Both strengths and limitations existed in our study. Strengths included a large number of samples, high event rates and a long-term follow-up (8 years). Many detailed clinical and biochemical assessments were included which allowed us to adjust for multiple potential confounders. After consulting numerous references, when known risk factors were controlled, our research is the first to report that TG level is a true independent risk factor associated with T2DM. Moreover, the same correlation between TG and T2DM in CHARLS supported this conclusion. It could provide a new approach to diagnose T2DM in clinical. As for limitations, one of the limitations was that the data of lifestyle choices such as drinking habits and smoking was not included in our study, which may affect the result [30, 31]. Another limitation was that our subjects were “relatively healthy”. These are people who had routine physical examinations and were not medical-seeking patients. While Tianjin General Hospital is a top tertiary care center, the subjects we included tended to be college teachers and/or staff of government-funded agencies. Our test population had relatively high socioeconomic status as a whole. Thus, our result might not represent the general population. As a strength, meanwhile, the long-term follow-up may be a limitation. Medicine testes were updated and new physical indicators were joined during the past 8 years, it made our cohort difficult to keep some indicators unbroken. We only have recent data for HbA1c (2014–2018) (Additional file 1: Table S2), the HbA1c level showed as an important indicator of T2DM (HR = 3.49, 3.21–3.80, *P *< 0.001) (Additional file 1: Table S3).

To conclusion, Both Tianjin General Hospital Cohort and CHARLS indicated that the increased TG level was significantly associated with incidence of T2DM. Higher TG level was associated with higher risk of T2DM independently. Survival analysis in Tianjin General Hospital Cohort showed that elevated TG level may predict higher cumulative incidence of T2DM. In order to decipher the relationship between TG and T2DM, future clinicals and basic research are needed.

## Supplementary information

**Additional file 1: Figure S1.** Flow chart of subject selection for the present study. **Figure S2.** TG levels and the time to onset for T2DM. **Table S1.** Baseline characteristics of subjects in CHARLS. **Table S2.** Baseline characteristics of subjects in Tianjin General Hospital Cohort from 2014–2018. **Table S3.** Hazard ratios of TG levels for T2DM in the Tianjin General Hospital Cohort.

## Data Availability

All the data and materials used in our article are available from the corresponding author on reasonable request.
