# Supplementary material for: Triglyceride is an independent predictor of type 2 diabetes among middle-aged and older adults: a prospective study with 8-year follow-ups in two cohorts
Source: J Transl Med. 2019 Dec 3;17:403. doi: 10.1186/s12967-019-02156-3 (PMC6894231; doi:10.1186/s12967-019-02156-3)
Supplement: Supplementary file 1 — Additional file 1: Figure S1. Flow chart of subject selection for the present study. Figure S2. TG levels and the time to onset for T2DM. Table S1. Baseline characteristics of subjects in CHARLS. Table S2. Baseline characteristics of subjects in Tianjin General Hospital Cohort from 2014–2018. Table S3. Hazard ratios of TG levels for T2DM in the Tianjin General Hospital Cohort. [file 12967_2019_2156_MOESM1_ESM.docx]

**Online-only Additional Material**

**Figure S1**

**Flow chart of subject selection for the present study.**


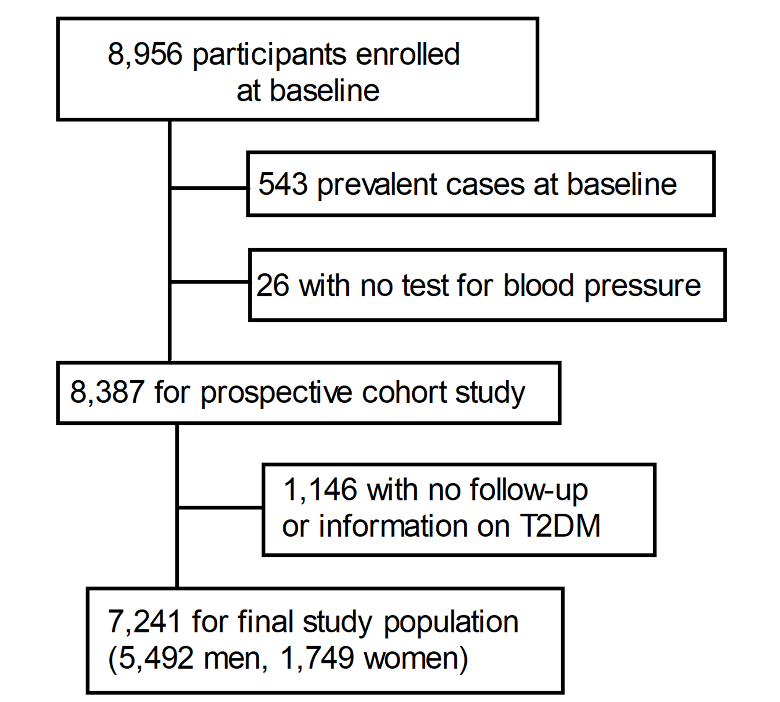


**Figure S2**

**TG levels and the time to onset for T2DM**


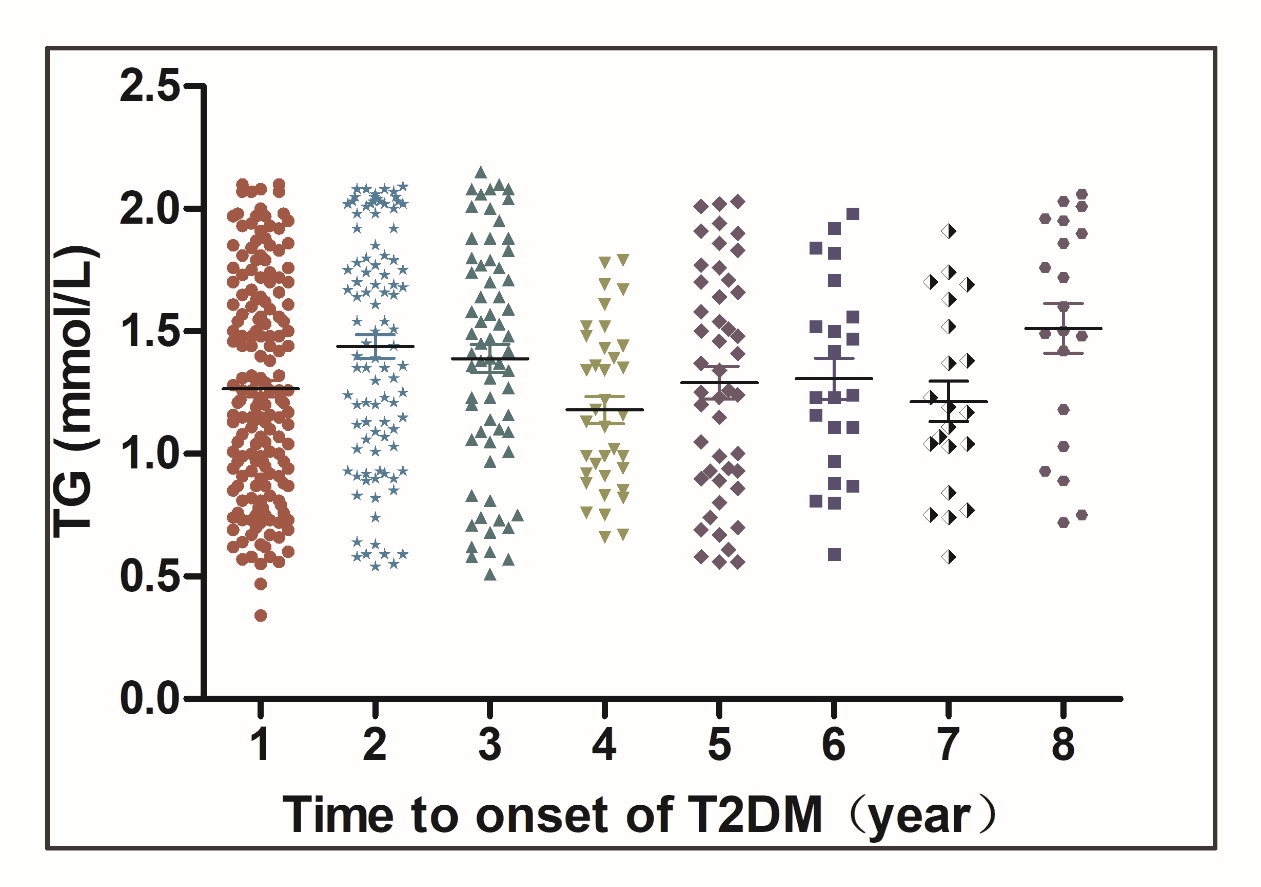


**Table S1**

**Baseline characteristics of subjects in CHARLS.**

| Characteristics | Normal  （＜1.7） | Borderline  （1.7-2.25） | High  （≥2.26） | *P*-value |
| --- | --- | --- | --- | --- |
| Male（%） | 3713/7102 (52.3%) | 763/1272 (60 %) | 710/1216 (58.4%) | 0.000 |
| Age（years） | 59.04±9.63 | 58.30±9.15 | 57.63±8.91 | 0.000 |
| BMI (kg/m²) | 22.94±3.46 | 24.60±3.47 | 25.21±3.60 | 0.000 |
| Hypertension (%) | 1149 (20.4) | 374 (29.4) | 390 (32.1) | 0.000 |
| TG (mmol/L) | 1.03±0.33 | 2.53±2.19 | 3.15±0.81 | 0.000 |
| TC (mmol/L) | 4.90±0.93 | 5.34±1.10 | 5.41±1.09 | 0.000 |
| Hyperuricemia | 542/7102 (7.6%) | 135/1272 (9.6%) | 196/1216 (16.1%) | 0.000 |

Date are expressed as mean ± SD, *n* (%). Abbreviations: BMI, body mass index; Hypertension; TG, plasma triglyceride level; TC, total cholesterol;

†Grouped based on TG level: normal ＜150mg/dL; borderline high 150 mg/dL-199mg/dL; high 200mg/dL-499mg/dL;

**P* < 0.05 was considered statistically significant

**Table S2**

**Baseline characteristics of subjects in Tianjin General Hospital Cohort from 2014-2018.**

| **Characteristics** | **Normal**  **(＜1.7)** | **Borderline**  **(1.7-2.25)** | **High**  **(≥2.26)** | ***P*-value** |
| --- | --- | --- | --- | --- |
| Male(%) | 3220（71.7%） | 763（75.8%） | 715（81.3%） | ＜0.001 |
| Age(years) | 66.53±15.20 | 64.16±13.98 | 59.93±14.17 | ＜0.001 |
| BMI(kg/m²) | 24.47±3.14 | 25.88±3.01 | 26.33±3.04 | 0.017 |
| Hypertension | 462（10.3%） | 128（12.7%） | 148（16.8%） | ＜0.001 |
| FPG(mmol/L) | 5.35±0.57 | 5.43±0.56 | 5.49±0.55 | ＜0.001 |
| TG(mmol/L) | 1.08±0.31 | 1.93±0.15 | 5.49±0.55 | ＜0.001 |
| TC(mmol/L) | 3.27±1.99 | 3.83±1.85 | 4.63±1.68 | ＜0.001 |
| Hyperuricemia | 597（13.3%） | 253（25.1%） | 326（37.1%） | ＜0.001 |
| SUA(μmol/L) | 325.36±76.18 | 362.85±78.90 | 389.24±83.56 | ＜0.001 |
| ALT(IU/L) | 18.10±12.17 | 23.56±16.23 | 27.65±18.07 | ＜0.001 |
| eGFR-ml/min/1.73² | 89.89±18.92 | 89.32±18.79 | 92.04±19.77 | 0.003 |
| HDL-C(mmol/L) | 1.42±0.38 | 1.16±0.26 | 1.06±0.24 | ＜0.001 |
| LDL-(mmol/L) | 2.25±1.00 | 2.40±1.10 | 2.52±1.05 | ＜0.001 |
| HbAlc | 5.71±0.49 | 5.75±0.49 | 5.74±0.48 | 0.010 |

6,375 participants (male 4698, 73.7%; female 1677, 26.3%); mean age 65.25±15.05;

Abbreviations: BMI, body mass index; Hypertension; FPG, fasting plasma glucose; TG, plasma triglyceride level; TC, total cholesterol; eGFR, estimated glomerular filtration rate; hyperuricemia, SUA, serum uric acid; ALT, alanine aminotransferase; HDL-C, High density lipoprotein cholesterol; LDL-C, low density lipoprotein cholesterol; HbAlc, glycated hemoglobin

Date are expressed as mean ± SD, n (%).

†Grouped based on TG level：normal ＜150mg/dL; borderline high 150 mg/dL-199mg/dL; high 200mg/dL-499mg/dL;

**P*< 0.05 was considered statistically significant.

**Table S3**

**Hazard ratios of TG levels for T2DM in the Tianjin General Hospital Cohort.**

|  | ***HR*（95%*CI*）** | ***P-*value** |
| --- | --- | --- |
| logTG | 1.53（1.04-2.25） | 0.033 |
| Female | 0.69（0.54-0.87） | 0.002 |
| Age (ears) | 1.00（0.99-1.00） | 0.943 |
| BMI (kg/ m²) | 1.04（1.00-1.07） | 0.009 |
| Hypertension | 1.40（1.09-1.79） | 0.008 |
| HbAlc | 3.49（3.21-3.80） | 0.000 |

Abbreviations: TG, plasma triglyceride level; BMI, body mass index; HbAlc, glycated hemoglobin

†Each time the value of hazard ratios changes by one unit, the risk doubles.

**P*< 0.05 was considered statistically significant.
